# 2198. Healthcare Utilization During Acute Medically Attended Respiratory Syncytial Virus (RSV) Episodes among Infants in the United States

**DOI:** 10.1093/ofid/ofac492.1817

**Published:** 2022-12-15

**Authors:** Jason Gantenberg, Robertus van Aalst, David R Diakun, Brendan L Limone, Christopher B Nelson, David A Savitz, Andrew R Zullo

**Affiliations:** Department of Health Services, Policy & Practice, Brown University School of Public Health, Providence, Rhode Island; Sanofi Vaccines, Lyon, Auvergne, France; IBM Watson Health, Cambridge, Massachusetts; IBM Watson Health, Cambridge, Massachusetts; Sanofi, Swiftwater, Pennsylvania; Brown University School of Public Health, Providence, Rhode Island; Brown University, Providence, Rhode Island

## Abstract

**Background:**

Healthcare utilization during acute medically attended (MA) RSV-associated lower respiratory tract infection (LRTI) episodes remains poorly characterized, particularly among term infants without comorbidities. Describing the care incurred during these episodes may provide important information regarding the impact of MA RSV LRTI.

**Objective:** Estimate the occurrence and average number of outpatient, emergency department (ED), and inpatient visits during infants' first RSV season.

**Methods:**

Using deidentified insurance claims data (MarketScan Commercial ®, MSC; MarketScan Medicaid ®, MSM; Optum Clinformatics ®, OC), we assembled a cohort of infants born in the United States between April 1, 2016 and February 29, 2020 and identified their first MA RSV LRTI episode during their first RSV season. We defined an RSV episode as the 7 days following an index RSV diagnosis (inclusive of the diagnosis date), allowing for two alternative definitions of the index diagnosis—a *specific* definition, based on ICD-10 codes explicitly indicating RSV, and a *sensitive* definition, including codes for unspecified bronchiolitis. We calculated the average number of outpatient, ED, and inpatient visits during this episode, stratifying estimates by gestational age and the presence/absence of comorbidities. We also calculated the proportion of episodes involving a given place of service.

**Results:**

Using the specific (sensitive) definitions, infants averaged 1.11 (1.19), 0.90 (0.89), and 1.56 (1.39) outpatient visits during their first acute RSV episode, in the MSC, MSM, and OC datasets, respectively (Table). They averaged 0.35 (0.23), 0.51 (0.47), and 0.39 (0.23) visits to the ED and 0.28 (0.14), 0.25 (0.14), and 0.23 (0.11) inpatient stays. While MA RSV LRTI episodes among infants who were preterm and/or had other comorbidities (comorbidity groups B and C) were more likely to involve an ED or inpatient visit, up to 21% (10%) of episodes among otherwise healthy term infants involved an inpatient stay.

**Figure:**
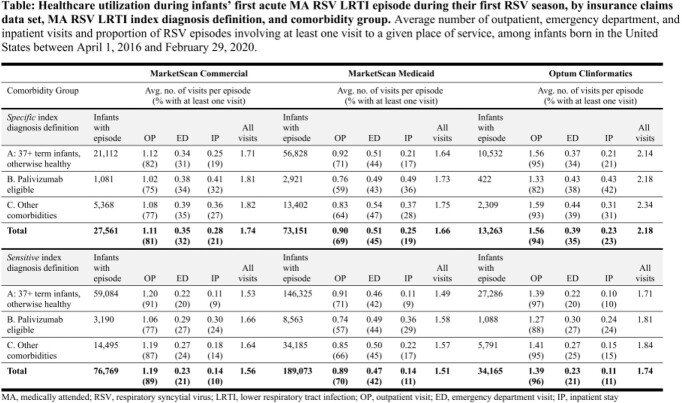

**Conclusion:**

Up to 1 in 5 infants experiencing an MA RSV LRTI episode during their first RSV season visited an inpatient setting, including between 9% and 21% of otherwise healthy term infants.

*This study was funded by Sanofi and AstraZeneca.*

**Disclosures:**

**Jason Gantenberg, PhD, MPH**, Sanofi: Grant/Research Support **Robertus van Aalst, PhD, MSc**, Sanofi: Stocks/Bonds **David R. Diakun, BS**, Sanofi: Employed by IBM Watson Health which was contracted by Sanofi to perfom outcomes research|Sobi: Employed by IBM Watson Health which was contracted by Sobi to conduct the study **Christopher B. Nelson, PhD MPH**, Sanofi: employee|Sanofi: Stocks/Bonds **David A. Savitz, PhD**, Sanofi-Pasteur: Grant/Research Support|Sanofi-Pasteur: Honoraria **Andrew R. Zullo, PharmD, PhD**, Sanofi: Grant/Research Support.

